# Candidate microRNA biomarkers of pancreatic ductal adenocarcinoma: meta-analysis, experimental validation and clinical significance

**DOI:** 10.1186/1756-9966-32-71

**Published:** 2013-09-28

**Authors:** Ming-Zhe Ma, Xiang Kong, Ming-Zhe Weng, Kun Cheng, Wei Gong, Zhi-Wei Quan, Cheng-Hong Peng

**Affiliations:** 1 Department of General Surgery, Xinhua Hospital, Shanghai Jiaotong University School of Medicine, 1665 Kongjiang Road, Shanghai 200092, People’s Republic of China; 2 Department of Endocrinology, Xinhua Hospital, Shanghai Jiaotong University School of Medicine, Shanghai, China; 3 Department of Pharmacology, Wannan Medical College, Anhui, China; 4 Department of General Surgery, Ruijin Hospital, Shanghai Jiaotong University School of Medicine, Shanghai, China

**Keywords:** microRNA, Meta-analysis, Pancreatic cancer, Biomarker

## Abstract

**Background:**

The diagnostic and prognostic value of microRNA (miRNA) expression aberrations in pancreatic ductal adenocarcinoma (PDAC) has been studied extensively in recent years. However, differences in measurement platforms and lab protocols as well as small sample sizes can render gene expression levels incomparable.

**Methods:**

A comprehensive meta-review of published studies in PDAC that compared the miRNA expression profiles of PDAC tissues and paired neighbouring noncancerous pancreatic tissues was performed to determine candidate miRNA biomarkers for PDAC. Both a miRNA vote-counting strategy and a recently published Robust Rank Aggregation method were employed. In this review, a total of 538 tumour and 206 noncancerous control samples were included.

**Results:**

We identified a statistically significant miRNA meta-signature of seven up- and three down-regulated miRNAs. The experimental validation results showed that the miRNA expression levels were in accordance with the meta-signature. The results from the vote-counting strategy were consistent with those from the Robust Rank Aggregation method. The experimental validation confirmed that the statistically unique profiles identified by the meta-review approach could discriminate PDAC tissues from paired nonmalignant pancreatic tissues. In a cohort of 70 patients, the high expression of miR-21 (*p*=0.018, HR=2.610; 95% CI=1.179-5.777) and miR-31 (*p*=0.039, HR=2.735; 95% CI=1.317-6.426), the low expression of miR-375 (*p*=0.022, HR=2.337; 95% CI=1.431-5.066) were associated with poor overall survival following resection, independent of clinical covariates.

**Conclusions:**

The identified miRNAs may be used to develop a panel of diagnostic and prognostic biomarkers for PDAC with sufficient sensitivity and specificity for use in a clinical setting.

## Introduction

Pancreatic ductal adenocarcinoma (PDAC) is one of the most aggressive of all malignancies [1]. Less than 20% of PDAC patients present with localised, potentially curable tumours. The overall 5-year survival rate is <5%. Because the chemotherapeutic options prolong life only minimally, the current PDAC mortality is nearly identical to its incidence [2]. Extensive studies have been performed to identify biomarkers for this disease. At the messenger RNA (mRNA) level, quite a few, including some very specific molecular variations have been found in cancerous tissues [3]. MicroRNAs (miRNAs), a class of short non-coding RNA molecules that range in size from 19 to 25 nucleotides, have been proposed as promising biomarkers of early cancer detection and accurate prognosis as well as targets for more efficient treatment [4,5]. MiRNAs play important roles in regulating the translation of many genes and the degradation of mRNAs through base pairing to partially complementary sites, predominately in the 3′ untranslated region [6,7]. Several studies have implicated miRNAs in the regulation of tumour biology [8-10]. Model biomarkers should be easily quantifiable and associate strongly with clinical outcome, and miRNAs may match these criteria.

High-throughput technologies have been employed to identify differences in miRNA expression levels between normal and cancerous tissues. These studies have the potential to identify dozens or hundreds of differentially expressed miRNAs, although only a small fraction of them may be of actual clinical utility as diagnostic/prognostic biomarkers. Finding a meaningful way in which to combine different data sources is often a non-trivial task. Differences in measurement platforms and lab protocols as well as small sample sizes can render gene expression levels incomparable. Hence, it may be better to analyse datasets separately and then aggregate the resulting gene lists. This strategy has been applied to identify gene co-expression networks [11] and to define more robust sets of cancer-related genes [12,13] and miRNAs [14,15].

In the meta-review approach, the results of several individual studies are combined to increase statistical power and subsequently resolve any inconsistencies or discrepancies among different profiling studies. In this study, we applied two meta-review approaches: the well-known vote-counting strategy [12,13], which is based on the number of studies reporting a gene as being consistently expressed and then further ranking these genes with respect to total sample size and average fold-change, and the recently published Robust Rank Aggregation method [16,17]. Pathway analysis was then performed to identify the physiological impact of miRNA deregulation in PDAC progression. Moreover, we further validated the most up-regulated and down-regulated miRNAs from the meta-review in a clinical setting. The expression levels of a subset of candidate miRNAs were assessed by quantitative real-time polymerase chain reaction (qRT-PCR). With the validation of candidate miRNAs, we selected the most promising miRNAs based on factors such as fold-change to explore their potential effects on the survival of PDAC patients after surgical resection.

## Materials and methods

### Selection of studies and datasets

The Scopus database (http://www.scopus.com) was searched for PADC miRNA expression profiling studies using search term TITLE-ABS-KEY [(mirna* OR microrna* OR mir-* OR mir) AND profil* AND (pancreas* cancer OR pancreatic carcinoma OR pancreas* neoplas* OR pancreatic tumo* OR carcinoma of pancreas* OR cancer of pancreas*)]. The same strategy was also applied to searches of the Gene Expression Omnibus (GEO; http://www.ncbi.nlm.nih.gov/geo/), ArrayExpress (http://www.ebi.ac.uk/arrayexpress/), and PubMed (http://www.ncbi.nlm.nih.gov/pubmed). The last search was performed on May 11, 2013. The titles and abstracts of the articles were screened, and the full text of the articles of interest was evaluated. We included only original experimental articles that were published in English and that compared the expression of miRNAs in PDAC tissue and noncancerous pancreatic tissue in humans. Articles were excluded based on the following criteria: (i) review articles, case reports or letters; (ii) non-English articles; (iii) studies of individual pre-selected candidate miRNAs; (iv) studies that used RT-PCR for initial selection (the reasons for this exclusion criterion are explained in the Discussion section); (v) studies using cell lines or serum from PDAC patients; (vi) studies that did not use a miRNA microarray platform; (vii) studies profiling different histological subtypes; (viii) studies that did not include noncancerous tissue.

### Data extraction

Two investigators (MM and XK) independently evaluated and extracted the data using standard protocols, and all discrepancies were resolved by a third investigator (MW). From the full text and corresponding supplemental information, the following eligibility items were collected and recorded for each study: author, region, period, selection and characteristics of the recruited PDAC patients, platform of miRNA expression profiling, and the list of up- and down-regulated miRNAs and their corresponding fold-changes. When the gene list was not available, the authors were contacted directly. All miRNA names were standardised according to miRBase version 20.

### Data processing

#### Vote-counting strategy

The miRNAs were ranked according to their importance as follows: (i) number of comparisons in agreement (i.e., listing the same miRNAs as having a consistent direction of change and being differentially expressed, respectively); (ii) total number of samples for comparison in agreement; (iii) average fold-changes reported for comparisons in agreement. Total sample size was considered more important than average fold-change because many studies did not report a fold-change. Furthermore, the average fold-change was based solely on the subset of studies for which a fold change value was available.

#### Robust rank aggregation method

The list of extracted miRNAs was ranked based on their associated *p*-values (less than 0.05 was considered significant) when their fold-changes were not reported. All of the protocols for the Robust Rank Aggregation method are free to download at the comprehensive R Archive Network website (http://cran.r-project.org/). Details can be found in the package documentation (http://cran.r-project.org/web/packages/RobustRankAggreg/RobustRankAggreg.pdf). This method assigns a *p-*value to each element in the aggregated list, which indicates how much better it is ranked compared with a null model, expecting random ordering. To assess the stability of the acquired *p-*values, leave-one-out cross-validation was applied in the Robust Rank Aggregation algorithm. This analysis was repeated 10,000 times, and each time, one random gene list was left out of the analysis. The *p-*values acquired from each round for each miRNA were then averaged.

### MiRNA target prediction and enrichment analysis

The mRNA targets of the miRNA genes were predicted using TargetScan (http://www.targetscan.org/), miRDB (http://mirdb.org/miRDB/), and miRANDA (http://www.microrna.org/microrna/getGeneForm.do), as each algorithm determines target binding differently. We selected targets from the miRANDA/miSVR search with scores less than −1.25 for further analysis. Enrichment analyses for KEGG and Panther pathways and Gene Ontology terms were performed with the GeneCodis tool (http://genecodis.dacya.ucm.es/). The potential targets of each miRNA were used as input.

### Ethics statement

Ethical approval for this study was obtained from the Department of General Surgery of Ruijin Hospital at Shanghai Jiaotong University (Shanghai, China). All patients provided informed written consent for their tissues to be used for scientific research and to publish their case details.

### Sample collection

Seventy-eight PDAC tissue samples and neighbouring noncancerous pancreatic tissue samples (collected postoperatively from September 2010 to August 2011) used in this study were obtained from the Department of General Surgery of Ruijin Hospital at Shanghai Jiaotong University (Shanghai, China). The specimens were obtained from patients undergoing PDAC resection with curative intent. All diagnoses were based on pathological and/or cytological evidence. The histological features of the specimens were evaluated by a senior pathologist according to the WHO (World Health Organization) classification criteria. The tissues were obtained before chemotherapy and radiation therapy. Upon removal of the surgical specimen, research personnel immediately transported the tissue to the surgical pathology lab. Pathology faculty performed a gross analysis of the specimen and selected pancreatic tissues that appeared to be cancerous and pancreatic tissues that appeared to be normal for analysis. Each sample was immediately frozen in liquid nitrogen and stored at −80°C prior to RNA isolation and qRT-PCR analysis. A second level of quality control was performed on the adjacent benign tissues. Histological slides were prepared from the section of frozen tissue that was directly adjacent to the tissue from which the RNA was isolated. These slides were examined by experienced pathologists to determine if the benign tissues contained any pancreatic tumour cells. Benign tissues that contained residual tumour tissues were not included in the study. Complete clinicopathological follow-up data of the PDAC patients from which the specimens were collected were available.

### Validation of the most up-regulated or down-regulated miRNAs using qRT-PCR

Total RNA was isolated from the frozen tissue sample with TRIzol (Invitrogen) according to the manufacturer’s instructions. First-strand complementary DNA (cDNA) was synthesised from 2 μg of the total RNA using an oligo-dT primer and superscript II reverse transcriptase (Invitrogen). Then, quantification of the most up-regulated or down-regulated miRNAs was performed by qRT-PCR using SYBRR Premix Ex Taq (TakaRa). The U6 primers were obtained from TakaRa. PCR was performed in a real-time PCR system (Bio-Rad) as follows: 95°C for 3 min, followed by 35 cycles of 95°C for 5 sec, 60°C for 20 sec and 72°C for 30 sec, and then 94°C for 1 min and 60°C for 1 min, with an increase of 0.5°C per cycle. The expression level values were normalised to those of the small nuclear RNA U6 as a control. Relative fold-changes of miRNA expression were calculated using the △△CT method, and the values were expressed as 2^-△△CT^. The primer sequences were as follows: U6, 5′-CTCGCTTCGGCAGCACA-3′ (forward), 5′-AACGCTTCACGAATTTGCGT-3′ (reverse); miR-155, 5′-cgGCGGTTAATGCTAATCGTG-3′ (forward), 5′-GTGCAGGGTCCGAGGT-3′ (reverse); miR-100, 5′-GAATTCCCATACTGGTTGGCTCCCGC-3′ (forward), 5′-CTCGAGACGAATTCAATCGAAATATTC-3′ (reverse); miR-21, 5′-ACACTCCAGCTGGGTAGCTTATCAGACTGA-3′ (forward), 5′-TGGTGTCGTGGAGTCG-3′ (reverse); miR-221, 5′-CCCAGCATTTCTGACTGTTG-3′ (forward), 5′-TGTGAGACCATTTGGGTGAA-3′ (reverse); miR-31, 5′-ACGCGGCAAGATGCTGGCA-3′ (forward), 5′-CAGTGCTGGGTCCGAGTGA-3′ (reverse); miR-143, 5′-CCTGGCCTGAGATGAAGCAC-3′ (forward), 5′-CAGTGCTGGGTCCGAGTGA-3′ (reverse); miR-23a, 5′-CTTGAACTCCTGGCCTGAAG-3′ (forward), 5′-GCCAAAGAAACACTCACAGCT-3′ (reverse); miR-217, 5′-GCGTACTGCATCAGGAACTGATTGGA-3′ (forward), 5′-GGGCACACAAAGGCAACTTTTGT-3′ (reverse); miR-148a, 5′-TCAGTGCACTACAGAACTTTGT-3′ (forward), 5′-GCTGTCAACGATACGCTACGT-3′ (reverse); miR-375, 5′-GAAGATCTTGAGGTACATCGCAGAGGCCAG-3′ (forward), 5′-CATGCCATGGGGGCCGGAGCGGAAGACCC-3′ (reverse).

### Statistical analysis

Kaplan-Meier survival analysis was used to analyse the association between postoperative survival and the miRNA expression level measured by qRT-PCR, and the resulting curves were divided into two classes (high and low expression in comparison to the mean level of miRNA expression as the threshold). Survival analysis was performed for each clinical covariate to assess their influence on outcome using a log-rank test. A multivariate Cox regression model was used to adjust for competing risk factors, and the hazard ratio (HR) with a 95% confidence interval (CI) was reported as an estimate of overall survival risk. The variables that were found to be significant in univariate analysis at *p*<0.05 were included in the final multivariate analysis in a backwards stepwise fashion. The statistical analyses were performed using the SPSS 18.0 for Windows software package (SPSS Inc.). Differences were considered to be statistically significant when the *p*-value was <0.05.

## Results

**Figure 1:**
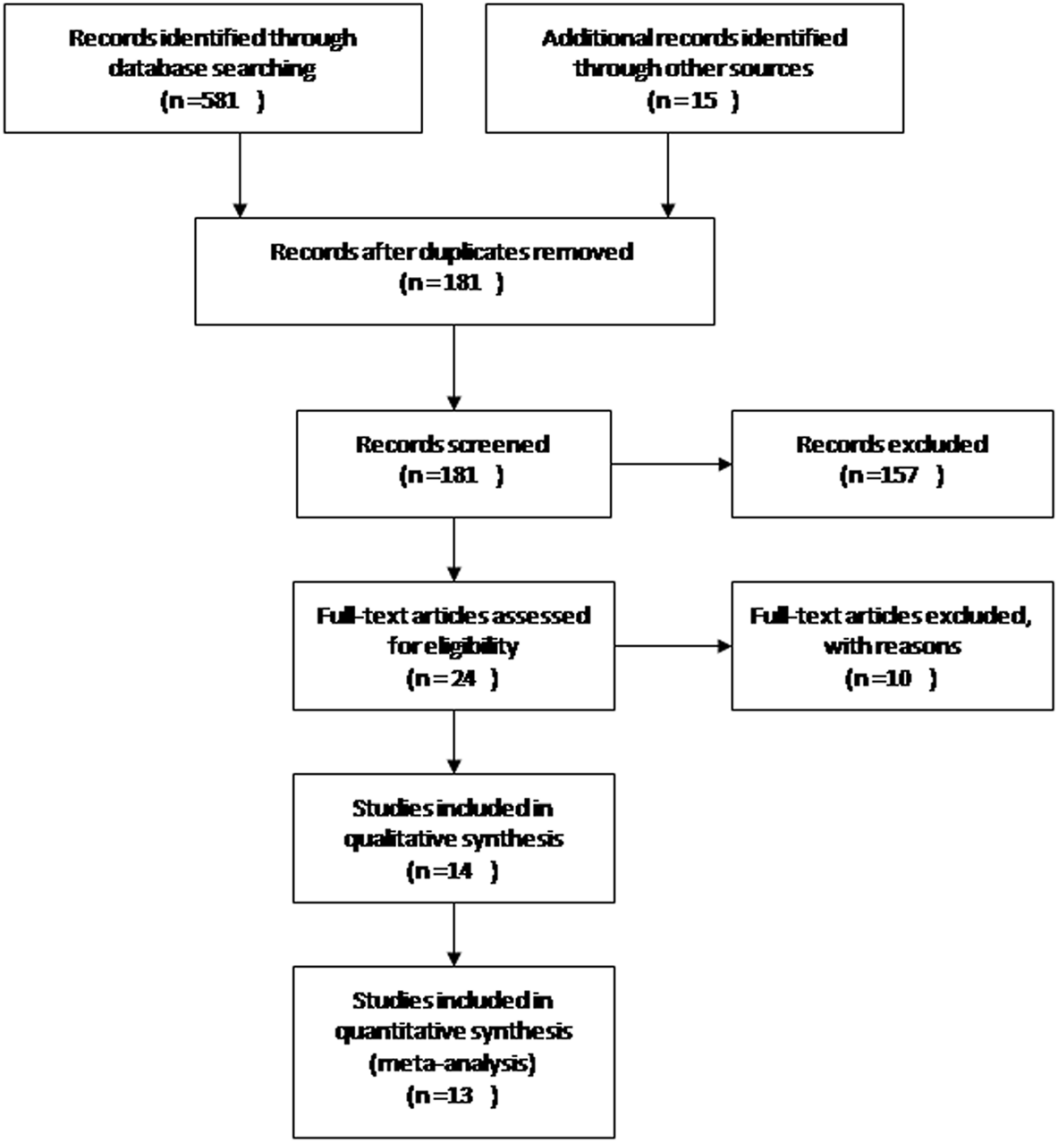
**PRISMA 2009 flow chart.** Only original experimental articles that were published in English and that analysed the differences in miRNA expression between PDAC tissue and noncancerous pancreatic tissue in humans were included. Articles were excluded if the studies did not use a miRNA microarray platform or if they profiled miRNAs in different histological subtypes.

Five hundred and ninety-eight relevant publications were indexed in the databases mentioned above (Scopus, GEO, PubMed and ArrayExpress). According to the inclusion criteria and the identification of duplicate publications, only fourteen independent studies [18-31] were included. However, one article was excluded for the unavailability of a ranked gene list both publically and in response to a request from the corresponding author [18]. The selection process is shown in Figure 1. Among the analysed studies, some of the studies employed patient samples as low as 5 [19] or 3 [20], which was too small to provide any reliable data. Not surprisingly, these two studies [19,20] were the basis for excluding numerous candidates that were consistently reported as either up- or down-regulated in other studies. The most glaring example of the strategic error of including these two studies in our meta-analysis is miRNA-100, which, despite being reported to be up-regulated in 7 studies [21,23,24,26,28,29,31], was considered to be down-regulated in one of the aforementioned studies [19], which only employed 5 tumour samples. Therefore, if Ref 19 was included, miR-100 would be listed as a miRNA with an inconsistent direction and would be subsequently excluded from the list of most consistently reported miRNAs. In addition, the fold-change in this study [19] was very low (less than 2) and may not have been significant if a large sample size was analysed. Other examples include miR-145, miR-141, miR-379, miR-200c, and miR-125b, which were reported in an opposite direction solely in these two studies. To avoid these deviations, these two small-sample-size studies were excluded from our meta-analysis. A brief description of the eleven included studies [21-31] and the acronyms by which the studies are referred to in the following text are provided in Table 1.

**Table 1:** Eleven microarray-based miRNA expression profiling studies of human PDAC tissues

**First author (reference)**	**Acronym**	**Region**	**Assay type**	**No. of probes**	**No. of samples (cancer/normal)**
AE Szafranska [21]	AE	USA	Custom microarray	377	13 (8/5)
Ada Piepoli [22]	AP	Italy	Affymetrix GeneChip array	866	NR (cancer=17)
Andrea S.Bauer [23]	AS	Germany	Geniom biochip miRNA homo sapiens	NR	110 (94/16)
Eun Joo Lee [24]	EJ	USA	Agilent Human miRNA Microarray	NR	28 (15/13)
Mark Bloomston [25]	MB	USA	Custom miRNA microarray	1100	130 (65/65), 65 pairs
Nicolai A.Schultz [26]	NA	Denmark	TaqMan array human microRNA A+B Cards v2.0	664	188 (160/28)
Nigel B.Jamieson [27]	NB	USA	Agilent Human miRNA Microarray (version 2.0)	723	58 (48/10)
Nicole C.Panarelli [28]	NC	USA	FlexmiR miRNA microarray	328	27 (17/10)
S Ali [29]	SA	USA	LC Science Houston microarray	NR	44 (29/15)
Shuyu Zhang [30]	SZ	China	Exiqon miRCURY LNA array	1200	40 (20/20), 20 pairs
Yuichi Nagao [31]	YN	Japan	Toray 3D-Gene miRNA microarray	>900	79 (65/24)

Abbreviations: *NR* not reported, pairs, cancerous and normal samples from the same patient.

The number of patients with PDAC that were investigated in these eleven studies ranged from 8 to 160 (median 47). The studies employed a diversity of microarray platforms (either commercial or custom), and the average number of miRNAs assayed was 778 (ranging from 377 to 1200; data were missing in three papers [23,24,29]). Only five studies [21-23,26,27] provided the whole list of differentially expressed miRNAs, while the others presented only a portion of their data. Our pooled dataset included a total of 538 tumour samples and 206 noncancerous control samples (at least), as in some studies, the number of noncancerous control samples was not specified [22].

A total of 439 differentially expressed miRNAs were reported in the eleven miRNA expression profiling studies; 254 were up-regulated and 185 were down-regulated in at least one study. Among the 439 miRNAs, 98 were reported in at least two studies; 77 (78.57%) with a consistent direction (Tables 2 and 3) and 21 with an inconsistent direction (Table 4) among the studies. Among the 77 miRNAs with a consistent direction, 50 were reported to be up-regulated (Table 2) and 27 were reported to be down-regulated (Table 3). One miRNA (miR-155) was reported in eight studies, three miRNAs (miR-21, miR-100 and miR-221) were reported in seven studies and twelve miRNAs were reported in at least five studies, with a consistent direction in all reports (Table 5). The miRNAs that were consistently reported in at least five studies are shown in Table 5. Although there were no strong disagreements between the individual miRNA profiling studies, the top lists varied considerably from study to study.

**Table 2:** Up-regulated miRNAs (n=50) reported in at least two expression profiling studies

**miRNA name**	**Studies with the same direction (reference)**	**No. of tissue samples tested**	**Mean fold-change**	**Mean rank**
hsa-miR-155	AE, AP, AS, EJ, MB, NB, NC, YN	329	4.98	12.62
hsa-miR-21	AE, MB, NA, NB, NC, SZ, YN	376	2.95	12.29
hsa-miR-100	AE, AS, EJ, MB, NB, NC, YN	317	8.07	13.00
hsa-miR-221	AE, AP, AS, EJ, MB, NB, NC	264	6.71	11.42
hsa-miR-31	AE, AP, AS, NA, YN	344	5.44	10.00
hsa-miR-10a	AE, AS, MB, NB, YN	280	2.50	14.60
hsa-miR-23a	AE, AP, AS, MB, NB	229	3.46	22.60
hsa-miR-143	AE, AP, MB, NB, YN	203	4.03	9.40
hsa-miR-222	AE, AS, MB, NB, YN	199	2.77	11.20
hsa-miR-210	AE, AS, MB, NA	323	2.97	16.00
hsa-miR-125a-5P	AE, AP, AS, MB	184	2.98	22.50
hsa-miR-145	AE, AP, AS, NB	167	3.75	9.75
hsa-miR-181a	AS, MB, NB	207	4.83	13.33
hsa-miR-199a-3p	AP, AS, YN	176	3.59	9.33
hsa-miR-23b	AS, AP, MB	176	3.09	42.33
hsa-miR-181b	AE, AS, MB	167	2.71	14.67
hsa-miR-199b-3p	AE, AS, NB	159	3.83	14.33
hsa-miR-331-3p	AP, AS, NB	159	1.83	35.33
hsa-miR-150	AE, AS, NB	150	3.73	6.67
hsa-let-7i	AE, AS, NB	150	2.47	17.33
hsa-miR-214	AE, AS, NB	147	3.63	11.00
hsa-miR-1246	AP, AS, SA	140	3.37	42.67
hsa-miR-223	AE, MB, NB	121	3.71	6.67
hsa-miR-24	AE, AP, NB	70	2.50	26.67
hsa-miR-584	AS, NA	254	5.81	64.50
hsa-miR-886-5p	AS, NA	254	3.26	38.50
hsa-miR-205	MB, NA	225	11.04	12.50
hsa-miR-142-3p	NA, NB	208	4.17	23.50
hsa-miR-451	NA, SA	189	28.36	16.00
hsa-miR-939	AP, NA	177	4.76	22.50
hsa-miR-196b	AE, NA	173	11.93	3.00
hsa-miR-99a	AS, YN	159	2.07	60.00
hsa-miR-181c	AS, MB	159	4.49	9.50
hsa-miR-199a-5p	AS, NB	142	2.64	18.50
hsa-miR-505	AS, NB	142	1.87	34.50
hsa-miR-342-3p	AS, NB	142	1.67	55.50
hsa-miR-140-3p	AS, NB	142	1.58	61.00
hsa-miR-34a	AS, NB	142	1.31	56.50
hsa-miR-92a	AS, SA	123	6.64	10.00
hsa-miR-320a	AS, SA	123	2.05	28.50
hsa-let-7e	AP, AS	111	4.31	36.50
hsa-miR-92b	AP, AS	111	1.66	47.50
hsa-miR-224	AE, AS	102	1.32	59.00
hsa-miR-99b	AE, AS	102	1.31	53.50
hsa-miR-93	AE, AS	98	1.83	21.50
hsa-miR-125b-1	EJ, MB	80	12.62	16.50
hsa-miR-106b	AE, NB	61	1.33	36.00
hsa-miR-27a	AE, NB	49	2.70	22.00
hsa-miR-17	AP, SA	42	2.77	14.50
hsa-miR-125b	AE, AS	25	1.89	22.00

**Table 3:** Down-regulated miRNAs (n=27) reported in at least two expression profiling studies

**miRNA name**	**Studies with same direction (reference)**	**No. of tissue samples tested**	**Mean fold-change**	**Mean rank**
hsa-miR-217	AE, AS, NA, NB, YN	371	18.16	4.20
hsa-miR-148a	AE, AS, MB, NA, NB	371	8.03	7.00
hsa-miR-375	AE, AS, MB, NA, NB	371	4.86	9.40
hsa-miR-216b	AS, NA, NB, YN	363	53.44	6.25
hsa-miR-216a	AS, NA, NB, YN	363	30.17	2.25
hsa-miR-130b	AE, AS, NA, NB	310	6.17	12.25
hsa-miR-141	NB, SZ, AE, AS	170	2.81	15.25
hsa-miR-30a-3p	NA, NB, AE	212	2.71	30.67
hsa-miR-200c	AE, AS, NB	150	2.66	23.67
hsa-miR-30a-5p	AS, NB, AE	150	2.16	27.67
hsa-miR-29c	AE, AS, NB	150	1.94	27.33
hsa-miR-30d	AE, AS, NB	150	1.73	35.33
hsa-miR-30e	AS, NB, AE	150	1.57	38.30
hsa-miR-379	SZ, AE, AS	122	1.62	21.67
has-miR-193b-3p	NA, NB	208	6.67	20.50
hsa-miR-184	AS, YN	159	2.82	26.50
hsa-miR-338-5p	AS, NB	142	3.15	25.50
hsa-miR-182	AE, AS	102	2.88	15.50
hsa-miR-30b	AE, AS	102	2.25	17.00
hsa-miR-335	AE, AS	102	2.16	15.00
hsa-miR-200a	AE, AS	102	1.66	24.50
hsa-miR-200b	AE, AS	102	1.62	28.00
hsa-miR-30c	AS, AS	98	2.18	17.00
hsa-miR-148b	AE, MB	73	2.52	2.50
hsa-let-7f	AE, SA	37	13.05	20.00
hsa-let-7c	AE, SA	37	2.66	23.50
hsa-let-7b	AE, SA	37	1.97	25.00

**Table 4:** Differentially expressed miRNAs (n=21) with an inconsistent direction between two studies

** miRNA name**	**Direction of expression**	**Studies with same direction (reference)**	**No. of tissue samples tested**	**Mean fold-change**	**Mean rank**
hsa-miR-103	↑	AE, AP, AS, NB	167	2.72	87.00
	↓	SZ	20	1.73	5.00
hsa-let-7d	↑	EJ, AP	32	6.82	11.50
	↓	SA, AE	37	7.04	22.50
hsa-miR-26a	↑	AP	17	5.16	12.00
	↓	AE, AS, SA	131	4.38	30.67
hsa-miR-146a	↑	AE, AS	102	2.08	12.00
	↓	SA	29	3.03	9.00
hsa-miR-708	↑	AS, NA	254	3.15	43.50
	↓	NB	48	9.26	7.00
hsa-miR-345	↑	AS	94	1.45	85.00
	↓	EJ, NB	63	12.59	2.50
hsa-miR-376a	↑	EJ	15	7.79	17.00
	↓	AE, AS	102	1.43	28.00
hsa-miR-494	↑	NA	160	4.23	41.00
	↓	NB, AE	56	3.86	14.50
hsa-miR-423-5p	↑	SA	29	9.03	4.00
	↓	YN, NB	113	2.77	30.00
hsa-miR-365	↑	SZ	20	1.75	2.00
	↓	AE, AS	102	1.80	17.00
hsa-miR-130a	↑	NB	48	2.00	28.00
	↓	AE, AS	102	1.62	29.50
hsa-miR-132	↑	AS	94	2.59	18.00
	↓	SZ	20	3.05	1.00
hsa-miR-324-3p	↑	AS	94	1.95	39.00
	↓	NB	48	2.16	50.00
hsa-miR-501-5p	↑	AS	94	1.59	64.00
	↓	NB	48	2.02	52.00
hsa-miR-874	↑	AS	94	1.49	80.00
	↓	NB	48	2.20	47.00
hsa-miR-518d-3p	↑	AS	94	1.30	103.00
	↓	NA	160	15.35	9.00
hsa-miR-28-3p	↑	AS	94	1.28	104.00
	↓	NB	48	4.49	23.00
hsa-miR-648	↑	NA	160	8.63	16.00
	↓	NB	48	9.07	8.00
hsa-miR-575	↑	NA	160	7.52	22.00
	↓	NB	48	4.38	24.00
hsa-miR-877	↑	NA	160	4.03	43.00
	↓	NB	48	3.48	28.00
hsa-let-7g	↑	NB	48	2.44	21.00
	↓	AE	8	1.06	45.00

**Table 5:** PDAC meta-signature from the vote-counting strategy (reported consistently in at least five studies)

**miRNA name**	**No. of studies**	**Mean fold-change**	**Mean rank**
Up-regulated			
hsa-miR-155	8	4.98	12.62
hsa-miR-21	7	2.95	12.29
hsa-miR-100	7	8.07	13.00
hsa-miR-221	7	6.71	11.42
hsa-miR-31	5	5.44	10.00
hsa-miR-10a	5	2.50	14.60
hsa-miR-23a	5	3.46	22.60
hsa-miR-143	5	4.03	9.40
hsa-miR-222	5	2.77	11.20
Down-regulated			
hsa-miR-217	5	18.16	4.20
hsa-miR-148a	5	8.03	7.00
hsa-miR-375	5	4.86	9.40

Using the Robust Rank Aggregation method, we identified a statistically significant meta-signature of 7 up- and 3 down-regulated miRNAs in PDAC samples compared to noncancerous pancreatic tissues (Table 6). All meta-signature miRNAs that reached statistical significance after Bonferroni correction were reported by at least 5 datasets. Majority of the meta-signature miRNAs belong to the broadly conserved seed family (conserved across most vertebrates and bony fish).

**Table 6:** PDAC meta-signature from the Robust Rank Aggregation method

**miRNA name**	**Corrected *p*-value**	**Permutation *p*-value**	**No. of studies**
Up-regulated			
hsa-miR-155	6.17E-11	8.64E-13	8
hsa-miR-100	3.32E-09	7.01E-11	7
hsa-miR-21	2.75E-09	3.29E-11	7
hsa-miR-221	1.56E-08	9.34E-10	7
hsa-miR-31	1.44E-05	8.83E-07	5
hsa-miR-143	6.78E-04	4.56E-06	5
hsa-miR-23a	3.27E-03	5.09E-05	5
Down-regulated			
hsa-miR-217	7.56E-07	4.37E-09	5
hsa-miR-148a	2.00E-05	3.55E-07	5
hsa-miR-375	1.08E-03	8.70E-06	5

Our results from the vote-counting strategy were almost the same with those from the Robust Rank Aggregation method. The ten GO processes and pathways that were most strongly enriched with respect to the meta-signature miRNA candidates (miR-155, miR-100, miR-21, miR-221, miR-31, miR-143, miR-23a, miR-217, miR-148a and miR-375) are shown in Table 7.

**Table 7:** The ten most strongly enriched GO processes and pathways with respect to the meta-signature miRNA candidates


GO processes			
	Process	Hyp*	Genes
	0006355: regulation of transcription, DNA-dependent	1.94E-31	128
	0045944: positive regulation of transcription from RNA polymerase II promoter	2.21E-18	73
	0045893: positive regulation of transcription, DNA-dependent	7.64E-14	89
	0007275: multicellular organismal development	1.99E-13	57
	0007165: signal transduction	1.16E-10	69
	0007399: nervous system development	8.52E-10	74
	0006915: apoptotic process	1.76E-09	57
	0045892: negative regulation of transcription, DNA-dependent	4.03E-09	55
	0007155: cell adhesion	5.06E-08	90
	0007411: axon guidance	9.83E-08	24
KEGG Pathways			
	Pathway	Hyp*	Genes
	05200: Pathways in cancer	1.84E-05	33
	04010: MAPK signalling pathway	3.62E-05	31
	04144: Endocytosis	1.89E-04	19
	04510: Focal adhesion	2.34E-04	25
	04810: Regulation of actin cytoskeleton	4.11E-04	22
	04350: TGF-beta signalling pathway	8.67E-04	12
	04141: Protein processing in endoplasmic reticulum	2.19E-03	18
	04630: Jak-STAT signalling pathway	5.07E-03	15
	04310: Wnt signalling pathway	5.29E-03	14
	04520: Adherens junction	5.68E-03	10
Panther pathways			
	Pathway	Hyp*	Genes
	P00057: Wnt signalling pathway	6.66E-09	36
	P00012: Cadherin signalling pathway	8.93E-06	20
	P00018: EGF receptor signalling pathway	1.25E-04	18
	P00034: Integrin signalling pathway	4.11E-04	17
	P00021: FGF signalling pathway	8.83E-04	14
	P00047: PDGF signalling pathway	2.18E-03	13
	P00060: Ubiquitin proteasome pathway	2.67E-03	11
	P00048: PI3 kinase pathway	5.06E-03	8
	P00036: Interleukin signalling pathway	6.23E-03	11
	P04393: Ras pathway	7.82E-03	10

The number of predicted target genes in the process or pathway is shown. Hyp*: corrected hypergeometric *p*-value.

### Experimental validation of the expression levels of the most deregulated miRNAs in patients with PDAC

To determine if the ten most deregulated miRNAs from the meta-analysis (miR-155, miR-100, miR-21, miR-221, miR-31, miR-143, miR-23a, miR-217, miR-148a and miR-375) could be used as diagnostic biomarkers of PDAC, the expression levels of these miRNAs were compared between PDAC tissues and neighbouring noncancerous tissues by qRT-PCR analysis. The results showed that the expression levels of miR-155, miR-100, miR-21, miR-221, miR-31, miR-143 and miR-23a were increased, whereas the levels of miR-217, miR-148a and miR-375 were decreased in the PDAC tissues (all *p*<0.05). Detailed data are available in Table 8.

**Table 8:** Relative expression of miRNAs in PDAC compared with matched normal pancreatic tissue controls determined by qRT-PCR

**miRNA name**				
**Up-regulated**	**PDAC**	**N**	***p-*** **value**	**Fold-change**
miR-155	5.56±1.00	2.71±0.66	<0.001	2.11±0.41
miR-100	7.40±2.21	3.91±1.32	<0.001	2.00±0.51
miR-21	3.80±0.99	1.7±0.35	<0.001	2.25±0.44
miR-221	8.03±2.77	3.26±0.67	<0.001	2.53±0.84
miR-31	6.52±0.98	2.93±0.39	<0.001	2.12±0.47
miR-143	7.45±1.22	2.21±1.43	<0.001	2.94±0.74
miR-23a	7.80±1.18	3.44±0.73	<0.001	2.35±0.52
Down-regulated				
miR-217	2.88±1.15	10.35±3.68	<0.001	3.91±1.36
miR-148a	3.85±1.48	10.39±2.97	<0.001	2.86±0.77
miR-375	4.00±1.55	7.05±1.99	<0.001	1.76±0.36

Data are expressed as the mean ± SD. N: matched normal pancreatic tissue.

### Determination of prognostic significance of the candidate miRNAs in PDAC

The clinicopathological characteristics of 78 PDAC patients are shown in Table 9. The expression levels of individual miRNAs along with other well-known potential prognostic clinicopathological factors, such as histology, T category, lymph node metastasis, tumour size, perineural invasion, venous invasion and margin were included in a univariate analysis. With respect to the miRNA expression levels, for the up-regulated miRNAs, a fold-change of ≥2 was defined as high expression, and a fold-change of <2 was defined as low expression; for the down-regulated miRNAs, a fold-change of ≥2 was defined as low expression, and a fold-change of <2 was defined as high expression. Patients with advanced disease (UICC stage IV and concomitance of distant metastases) were excluded because we assumed that the prognosis of these patients (n=8) is determined by the occurrence of relapse or metastasis rather than other biological characteristics, such as miRNA expression levels.

**Table 9:** Clinicopathological characteristics of 78 PDAC patients

**Gender**	
Male	44 (56%)
Female	34 (44%)
T category	
T1	14 (18%)
T2	26 (33%)
T3	28 (36%)
T4	10 (13%)
N category	
NO	34 (44%)
N1	44 (56%)
M category	
M0	70 (90%)
M1	8 (10%)
Tumour size	
≥2 cm	42 (54%)
<2 cm	36 (46%)
Histology	
Well or moderately differentiated	38 (49%)
Poorly differentiated	40 (51%)
Perineural invasion	
None or slight	46 (59%)
Prominent	32 (41%)
Venous invasion	
None or slight	40 (51%)
Prominent	38 (49%)
Tumour grade (UICC)	
Stage I-IIA	32 (41%)
Stage IIB-IV	46 (59%)
Resection margin status	
R0	32 (41%)
R1	46 (59%)

**Figure 2:**
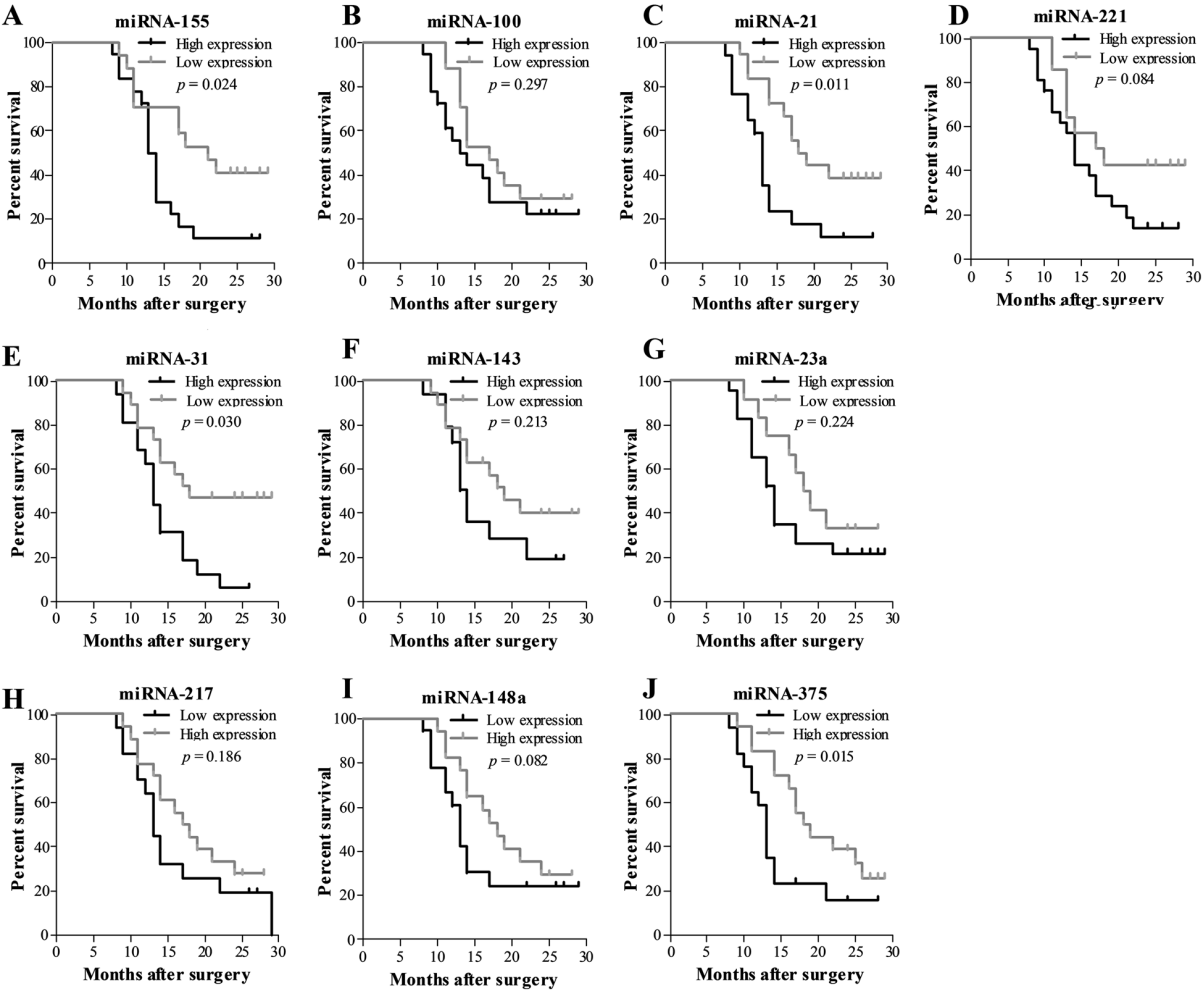
**Kaplan-Meier analysis of overall survival in patients with PDAC based on their expression of miR-155 (A), miR-100 (B), miR-21 (C), miR-221 (D), miR-31 (E), miR-143 (F), miR-23a (G), miR-217 (H), miR-148a (I) and miR-375 (J). ***p-*values are based on the log-rank test.

Kaplan-Meier survival analysis was used to analyse the association between postoperative survival and the miRNA expression level, and the resulting curves were divided into two classes (high and low expression in comparison with the mean level of miRNA expression as the threshold), as shown in Figure 2.

A univariate analysis using the Cox hazard regression model demonstrated that a high expression level of miR-21 (*p*=0.018, HR=2.610; 95% CI=1.179-5.777) and miR-155 (*p*=0.035, HR=2.414; 95% CI=1.064-5.478), a low expression level of miR-375 (*p*=0.022, HR=2.337; 95% CI=1.431-5.066), T category (*p*=0.039, HR=2.282; 95% CI=1.043-4.994) and margin involvement (*p*=0.026, HR=2.550; 95% CI=1.120-5.805) are associated with poor patient survival.

### Identification of two prognostic miRNAs by multivariate analysis

In a multivariate analysis using the Cox hazard regression model, a high expression level of miR-21 (*p*=0.021, HR=2.599; 95% CI=1.151-5.867), a low expression level of miR-375 (*p*=0.034, HR=2.451; 95% CI=1.429-5.135) and margin involvement (*p*=0.030, HR=2.543; 95% CI=1.093-5.918) were identified as significant unfavourable prognostic factors (Table 10).

**Table 10:** Univariate and multivariate survival analysis of the clinicopathological and molecular features of PDAC

**Factor**		**Univariate analysis**		**Multivariate analysis**	
		**HR (95% CI)**	***p*-value**	**HR (95% CI)**	***p*-value**
Histology	Well or moderate vs. poor	1.342 (0.621–2.901)	0.454		
T category	T 1/2 VS. T 3/4	2.282 (1.043–4.994)	0.039	1.518 (0.666–3.460)	0.320
Lymph node metastasis	Negative vs. positive	1.935 (0.867–4.317)	0.107		
Tumour size	<2 cm vs. ≥2 cm	1.736 (0.790–3.814)	0.170		
Perineural invasion	None or slight vs. prominent	1.244 (0.563–2.752)	0.589		
Margin involvement	R0 vs. R1	2.550 (1.120–5.805)	0.026	2.543 (1.093–5.918)	0.030
Vascular invasion	None or slight vs. prominent	2.542 (1.154–5.601)	0.021	1.940 (0.819–4.597)	0.132
miR-155 expression	High vs. low	2.414 (1.064–5.478)	0.035	1.365 (0.520–3.579)	0.538
miR-100 expression	High vs. low	1.480 (0.683–3.205)	0.321		
miR-21 expression	High vs. low	2.610 (1.179–5.777)	0.018	2.599 (1.151–5.867)	0.021
miR-221 expression	High vs. low	2.001 (0.868–4.617)	0.104		
miR-31 expression	High vs. low	2.735 (1.317-6.426)	0.039	2.637 (1.298-6.635)	0.048
miR-143 expression	High vs. low	1.516 (1.211–4.429)	0.257		
miR-23a expression	High vs. low	1.639 (0.709–3.788)	0.248		
miR-217 expression	Low vs. high	1.419 (1.045-4.021)	0.205		
miR-148a expression	Low vs. high	1.739 (1.385-4.481)	0.093		
miR-375 expression	Low vs. high	2.337 (1.431-5.066)	0.022	2.451 (1.429-5.135)	0.034

## Discussion

The common drawback of miRNA expression profiling studies is the lack of agreement among several studies. Differences in measurement platforms and lab protocols as well as small sample sizes can render gene expression levels incomparable. Sato et al. [32] and Wang et al. [33] systematically analysed representative miRNA profiling platforms and revealed that each platform is relatively stable in terms of its own intra-reproducibility; however, the inter-platform reproducibility among different platforms is low. Although the ideal method involves the analysis the raw miRNA expression datasets that are pooled together, such a rigorous approach is often impossible due to the unavailability of raw data and the low inter-platform concordance of results among different studies would bring difficulties to the analysis. To overcome these limitations, it might be better to analyse datasets separately and then aggregate the resulting gene lists. In this study, we used a meta-analysis approach to analyse PDAC-specific miRNAs derived from independent profiling experiments. The well-known vote-counting strategy [12,13] and the recently published Robust Rank Aggregation method [16,17] were employed. The core elements of the two methods were searches for the most recognised miRNAs among the included studies.

Two principal methods are used to measure miRNA expression levels: qRT-PCR and microarray hybridisation. The technological merits and drawbacks of qRT-PCR and microarrays for miRNA analysis are similar to those for RNA or genomic DNA quantification [34]. RT-PCR, a semiquantitative method, is labour intensive and provides data for only one, or very few, miRNA(s) per assay. However, the rapid increase in the number of known miRNAs renders this method inefficient on a genomic scale, and it is most likely better used as a tool for validation rather than discovery. Microarrays are the best option for a standardised genome-wide assay that is amenable to high-throughput application [35]. As qRT-PCR detects only preselected miRNAs, mostly the miRNAs that were shown to be differentially expressed in PDAC from normal tissue in other studies, it hinders the discovery of new miRNAs. Most importantly, the results of studies using qRT-PCR analysis [36-40] were consistent with those of microarray-based studies. In addition to the intra-platform deviations between microarray and qRT-PCR analyses [35], we excluded qRT-PCR-based studies and focused on studies using miRNA microarray platforms.

We identified a meta-signature of seven up- and three down-regulated miRNAs. To our knowledge, no meta-analysis of miRNA profiling studies has specifically investigated PDAC. Furthermore, this is the first study that used a combination of the two most commonly used methods in the meta-analysis of miRNA and gene profiling. To determine if the identified miRNAs could be used as diagnostic biomarkers, we experimentally validated the expression of these miRNAs in a set of PDAC samples.

There are several factors that must be considered when choosing miRNAs as candidate diagnostic biomarkers for PDAC. First, the fold-change of the biomarker should be significant enough to discriminate cancerous tissue from benign tissue. As is shown in Tables 2 and 3, the average fold changes of the 10 miRNAs identified in the microarray-based studies were all >2. In addition, the candidate miRNAs should be expressed in a majority of tissues. As was validated by qRT-PCR, the up-regulated miRNAs were all expressed in more than 85% of the samples tested (data not shown).

Second, the biological function of each individual miRNA should be thoroughly investigated. A single miRNA may have dozens of targets, and a specific mRNA may be regulated by multiple different miRNAs [7]. A better understanding of the targets of the miRNAs would advance their use in clinical settings. As shown in Table 7, the ten most strongly enriched GO processes and pathways with respect to the meta-signature miRNA candidates were identified. In the GO processes list, regulation of transcription, DNA-dependent; positive regulation of transcription from RNA polymerase II promoter; and positive regulation of transcription, DNA-dependent were ranked as the top three, which is in accordance with the known primary functions of miRNAs [6,7]. Pathways in cancer and Wnt signalling pathways were ranked first in the KEGG and Panther pathway lists, respectively, highlighting the essential roles of miRNAs in cancer development.

Third, there should be adequate information about the pattern of expression of the miRNAs in different types of specimens. It has been indicated that circulating miRNAs in plasma could be more tissue-specific than tumour-specific [41,42]. In the context of the vast inconsistency between tissue-based and plasma-based results [23], we focused on studies that analysed miRNA expression between PDAC tissues and noncancerous pancreatic tissues in humans.

Last but not least, rigorous validation and demonstration of reproducibility in an independent cohort of patients are necessary to confirm the diagnostic value of miRNAs. With this in mind, we experimentally validated 10 candidate miRNAs in PDAC samples and confirmed that these 10 miRNAs were differentially expressed between PDAC tissues and noncancerous pancreatic tissues.

Considering that miRNA expression is able to successfully discriminate normal from cancerous pancreatic tissues, it is tempting to speculate that miRNAs could also predict cancer prognosis. However, our results do not exclude the possibility that other miRNAs are associated with prognosis, as we only studied a meta-signature of 10 miRNAs in a limited number of PDAC samples (n=78). The main reason for the possible association between miRNAs not within this meta-signature and prognosis may centre on the relatively small sample size in our study and others [25,27]. It is quite unrealistic to include all the miRNAs in Kaplan-Meier survival analyses, as it would be very laborious and time-consuming. Thus, commonly, only the candidate miRNAs with the greatest fold changes are included. As mentioned above, although there were no strong disagreements between the individual miRNA profiling studies, the top lists varied considerably from study to study. To remedy this problem, it was critical to identify the most differentially expressed miRNAs. We used a meta-review approach, which combines the results of several individual studies to increase statistical power and to subsequently resolve the inconsistency among different profiling studies. A meta-signature of seven up- and three down-regulated miRNAs was identified. Then, in independent patient samples, miR-21, miR-31 and miR-375 were found to be associated with cancer prognosis.

From our point of view, great caution should be taken in future research in this field. To start, sample sizes should be increased to minimise random sampling error. Next, as it is impossible for every researcher to use the same platform, reliable microarray platforms should be employed in all experiments. Finally, it is advisable to obtain an integrated view of the candidate miRNAs from many studies to avoid one-sided opinions, as great discrepancies exist among the studies.

Our study presents a method to resolve the differences that exist among studies and might have some clinical significance for research on miRNAs in PDAC. The 10 identified miRNAs may be used as diagnostic biomarkers or even therapeutic targets. In addition to our meta-analysis, we performed further studies examining the expression of the candidate miRNAs in PDAC samples and confirmed miR-21, miR-31 and miR-375 as potential prognostic biomarkers for PDAC.

## Competing interests

The authors declare that they have no competing interests.

## Authors’ contributions

MZM, XK and MZW conceived the study and participated in the data collection and analysis. MZM, XK and MZW performed the experiments. MZM and KX analysed the data. MZM, XK, ZWQ, WG and CHP wrote the paper. All authors read and approved the final manuscript.
